# On the Vapor Trail: Examining the Chemical Content of E-Cigarette Flavorings

**DOI:** 10.1289/ehp.124-A115

**Published:** 2016-06-01

**Authors:** Carrie Arnold

**Affiliations:** Carrie Arnold is a freelance science writer living in Virginia. Her work has appeared in *Scientific American*, *Discover*, *New Scientist*, *Smithsonian*, and more.

In 2014 the *Oxford English Dictionary* named “vape” its word of the year, joining “selfie” and “binge-watching” in the modern lexicon,^1^ a testament to the growing popularity of electronic nicotine delivery systems, including electronic cigarettes. E-cigarettes use cartridges of “e-juice” to produce vapor in a variety of flavors. Researchers report in this issue of *EHP* that 92% of e-juice products tested contained at least one of three compounds implicated in occupational lung problems.^2^

As the popularity of e-cigarettes increases, so does the controversy regarding potential benefits and risks of using them. Advocates for their use in harm reduction, like David Nutt at Imperial College London, say e-cigarettes are safer than traditional tobacco cigarettes and thus represent a major public health advance.^3^ Others say this claim overlooks the possibility that flavored e-cigarettes may attract users who might not otherwise use tobacco.^4^ Two recent literature reviews concluded that smokers who reported using e-cigarettes were actually less likely to stop smoking than smokers who didn’t, contrary to popular belief.^5^ ^,^ ^6^

**Figure:**
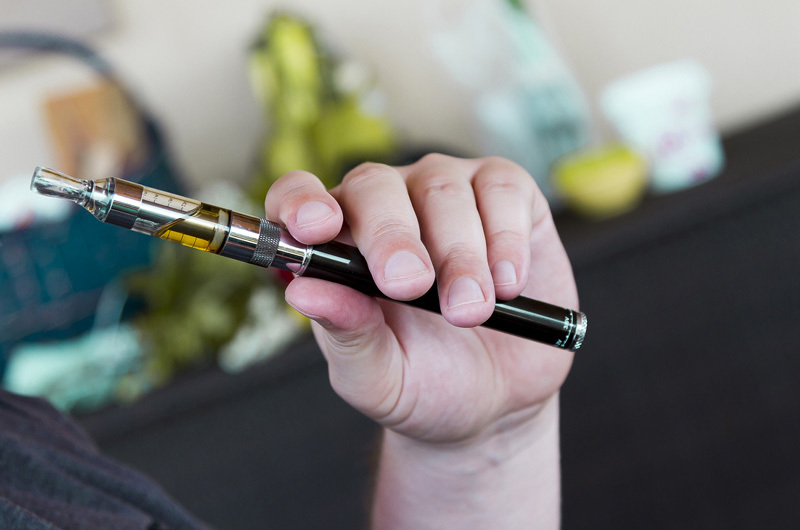
New findings suggest diacetyl and related chemicals could be widespread in e-juice, but it’s too soon to say how that may or may not translate into human health effects. © Adél Békefi/Getty

Until mid 2016, e-cigarettes were not regulated by the U.S. Food and Drug Administration or subject to age requirements placed on tobacco products by the Tobacco Control Act of 2009.^4^ Up to this point the devices enjoyed rapid growth among younger users. In 2015 16.0% of high schoolers and 5.3% of middle schoolers surveyed by the Centers for Disease Control and Prevention reported using e-cigarettes in the preceding 30 days, a 91% and 89% increase, respectively, since 2011.^7^

Many of the chemicals used to flavor e-juice, including diacetyl and the closely related compounds 2,3-pentanedione, and acetoin, have been used by the food industry for decades. However, the Flavor and Extract Manufacturers Association (FEMA) notes that none of the main safety assessment programs for flavors evaluate their use in products other than human food. FEMA further states that manufacturers and marketers must not claim that e-juice flavorings are safe simply because they have FEMA’s approval for use in food.^8^

Because inhalation of diacetyl and 2,3-pentanedione has been implicated in occupational lung disease in workers at a microwave popcorn plant^9^ and a coffee processing facility,^10^ many researchers believe it is prudent to assess potential exposures to these chemicals in e-cigarette users. For the current study, Joseph Allen, an environmental health scientist at Harvard University, and colleagues set out to find out how widespread diacetyl, 2,3-pentanedione, and acetoin were in samples of e-juice. They tested the vapor produced using 51 products, which came from leading brands or were deemed especially appealing to young users.

At least one of the compounds was found in 47 of the 51 flavors, including 39 containing diacetyl, 23 containing 2,3-pentanedione, and 46 containing acetoin. Concentrations of the three chemicals in individual samples ranged from barely detectable to 239 μg, 64 μg, and 529 μg, respectively, per e-cigarette.^2^

The 51 e-juices sampled make up a very small proportion of all the products sold, and there is variability in the chemical content of specific products as well as how those chemicals are delivered by different devices. The authors therefore acknowledge that it is impossible to extrapolate their results to all the other products on the market. Importantly, this study did not assess levels of diacetyl, 2,3-pentanedione, and acetoin in actual users, much less health effects. So it’s premature to assume that exposure to these chemicals via e-cigarettes causes health problems.

However, given the lack of testing, it’s also premature to assume the devices are completely safe. “There are many other worrisome chemicals besides these in e-cigarettes,” says James F. Pankow, an environmental health scientist at Portland State University. “We can’t do the toxicology work fast enough.” Besides, he says, the human response is very complicated, and it takes time for diseases to develop from chronic exposures: “We just don’t know what health effects will show up in epidemiological data 20 or 30 years from now.”
